# Evaluating the Clinical Use and Utility of a Digital Support App for Employees With Chronic Pain Returning to Work (SWEPPE): Observational Study

**DOI:** 10.2196/52088

**Published:** 2023-12-11

**Authors:** Christina Turesson, Gunilla Liedberg, Mathilda Björk

**Affiliations:** 1 Division of Prevention, Rehabilitation and Community Medicine Department of Health, Medicine and Caring Sciences Linköping University Linköping Sweden; 2 Pain and Rehabilitation Centre Department of Health, Medicine and Caring Sciences Linköping University Linköping Sweden

**Keywords:** chronic pain, digital support, eHealth, return-to-work, user data, mobile phone

## Abstract

**Background:**

The digital app SWEPPE (sustainable worker, a digital support for persons with chronic pain and their employers) was developed to improve the support of people with chronic pain in their return-to-work process after sick leave and includes functions such as the action plan, daily self-rating, self-monitoring graphs, the coach, the library, and shared information with the employer.

**Objective:**

This study aims to describe the use of the smartphone app SWEPPE among people with chronic pain who have participated in an interdisciplinary pain rehabilitation program.

**Methods:**

This is a case study including 16 people participating in a feasibility study. The analyses were based on user data collected for 3 months. Quantitative data regarding used functions were analyzed with descriptive statistics, and qualitative data of identified needs of support from the employer were grouped into 8 categories.

**Results:**

Self-monitoring was used by all participants (median 26, IQR 8-87 daily registrations). A total of 11 (N=16, 69%) participants set a work-related goal and performed weekly evaluations of goal fulfillment and ratings of their work ability. In total, 9 (56%) participants shared information with their employer and 2 contacted the coach. A total of 15 (94%) participants identified a total of 51 support interventions from their employer. Support to adapt to work assignments and support to adapt to work posture were the 2 biggest categories. The most common type of support identified by 53% (8/15) of the participants was the opportunity to take breaks and short rests.

**Conclusions:**

Participants used multiple SWEPPE functions, such as daily self-registration, goal setting, self-monitoring, and employer support identification. This shows the flexible nature of SWEPPE, enabling individuals to select functions that align with their needs. Additional research is required to investigate the extended use of SWEPPE and how employers use shared employee information.

## Introduction

### Background

Returning to work or staying at work can be challenging for people with chronic pain [1-3], and it can further make it difficult to handle the balance between working life and other daily activities [4]. Managing the needs of the person with chronic pain in relation to their colleagues and the organizations’ requirements also contributes to the complexity of creating a successful return-to-work (RTW) routine [2,5]. In addition, the experience of RTW may vary significantly between individuals who resume their duties in a physical office or workplace and those who can work from home, with less distress for remote workers [6]. Interprofessional rehabilitation programs (IPRPs) for people with chronic pain have shown positive outcomes in daily life functioning, life satisfaction, and pain [7]. However, people with chronic pain who have participated in an IPRP have also reported a lack of support and understanding from the employer when the responsibility for the RTW process is taken over by the employer. On the other hand, the employers described a lack of knowledge about how to support the employee with chronic pain [8]. Several studies have highlighted that a successful RTW process is characterized by a close collaboration and communication between the employer and employee [2,4,9]. Furthermore, taking the whole life situation into account for the person with chronic pain also plays an important part in the ability to participate in work and create a positive RTW [9,10].

Digital interventions and smartphone apps for the management of chronic pain is an evolving area [11]. It has been concluded that smartphone apps for pain management should be created using co-design involving developers, health care providers, and end users [12-14]. The efficacy of digital interventions has not yet been fully investigated [11], but web-based care for people with chronic pain has shown positive effects on pain levels and quality of life [15-17]. Positive results, such as reduction in care-seeking, disability, and pain, have also been reported in recent studies of mobile health (mHealth) apps for self-management of chronic pain [17-20]. mHealth apps have been used for identifying health needs among young people with chronic pain [21], self-monitoring to enhance adherence to treatment [22], or providing education and strengthening exercise therapy to increase work productivity [20]. However, there is a lack of mHealth apps or digital interventions targeting RTW for people with chronic pain and addressing the need for collaboration between the person and employer representative to create a successful RTW or staying at work.

To address the lack of support experienced by people with chronic pain and building on the technology of mHealth solutions, we developed the smartphone app SWEPPE (sustainable worker, a digital support for persons with chronic pain and their employers) through a user-centered agile design approach [23]. The intention was to create an eHealth tool with evidence-based content to enable sustainable RTW through collaboration between the employee with chronic pain and the employer. In total, 2 reference groups consisting of people with chronic pain and employers participated in the development and usability testing of SWEPPE. The development study found that SWEPPE supports individuals with chronic pain by offering user-friendly features such as goal setting, identifying barriers and RTW strategies, self-monitoring, and facilitating information sharing between employee and employer [23]. In SWEPPE, the person with chronic pain can use several features such as creating their own action plan for RTW, including setting a goal regarding employment rate and when to fulfill the goal. They can also perform self-rating and self-monitoring of health and work aspects, communicate with a coach, access a library with evidence-based knowledge, and share information with their employer. The acceptability of SWEPPE has been tested in a feasibility study [24], and a randomized controlled trial investigating the clinical effectiveness of SWEPPE is ongoing [25]. The feasibility study showed that patients and employers gained increased understanding and knowledge from using SWEPPE and found it supportive to set a work-related goal and to identify barriers and strategies for RTW. SWEPPE has also been found to contribute to improved collaboration between the employer and employee. However, there was a variation in the acceptability and experiences of using the different modules in SWEPPE, where high pain levels and low energy levels could be reasons for not using SWEPPE [24]. As there is a lack of knowledge about how different components of mHealth apps contribute to the self-management of pain [11,26], it can be valuable to analyze which and how different components are primarily used in mHealth support apps.

### Objective

This study aimed to describe the clinical use and utility of SWEPPE based on user data collected from people with chronic pain who used SWEPPE for 3 months after participating in an interdisciplinary pain rehabilitation program.

## Methods

### Study Design

This is the second part of the feasibility study of the digital support app SWEPPE. In the feasibility study, 16 patients with chronic pain (musculoskeletal pain for >3 mo) used SWEPPE for 3 months. The participants decided whether to invite their employers to the study, and 4 employers participated in the study. The first part of the feasibility study reports results from questionnaires and interviews with 11 patients and 4 employers regarding the acceptability of SWEPPE [24]. In this second part, we explore the utility of different functions in SWEPPE by analyzing user data collected in the SWEPPE database.

### Participants

Participants were recruited from health care units providing IPRPs for people with chronic pain in Sweden. To be included in the IPRPs in Sweden, the following criteria were used: persistent or intermittent pain lasting ≥3 months, pain largely affecting daily activities, completed systematic assessment and nonpharmacological optimization, and completed screening for psychosocial risk factors and differential diagnosis. The sample consisted of 16 people with chronic pain who had participated in the IPRPs at specialist or primary care clinics in southern Sweden. They were invited to participate in the study before the end of the IPRPs by the treating occupational therapists. An inclusion criterion was also being used, and they are currently on sick leave to some degree (25%-100%) with the aim of returning to their work. In total, 31 people met the inclusion criteria and agreed to receive more information about the study. A total of 8 people did not respond to the written information, and 6 declined participation in the study because they did not have the energy to learn something new, already had a plan for RTW, or perceived SWEPPE to be too demanding. In addition, 1 person was excluded at the start of the study because they no longer fulfilled the criteria of having employment. The remaining 16 people, which included 13 women and 3 men with a mean age of 35 (SD 5) years who gave informed consent to participate in the study and set up an account in SWEPPE. Examples of employment were teachers or teacher or student assistants, support assistants, nursery school workers, IT consultants, curators, and administrators.

### The SWEPPE Smartphone App Intervention

**Figure 1:**
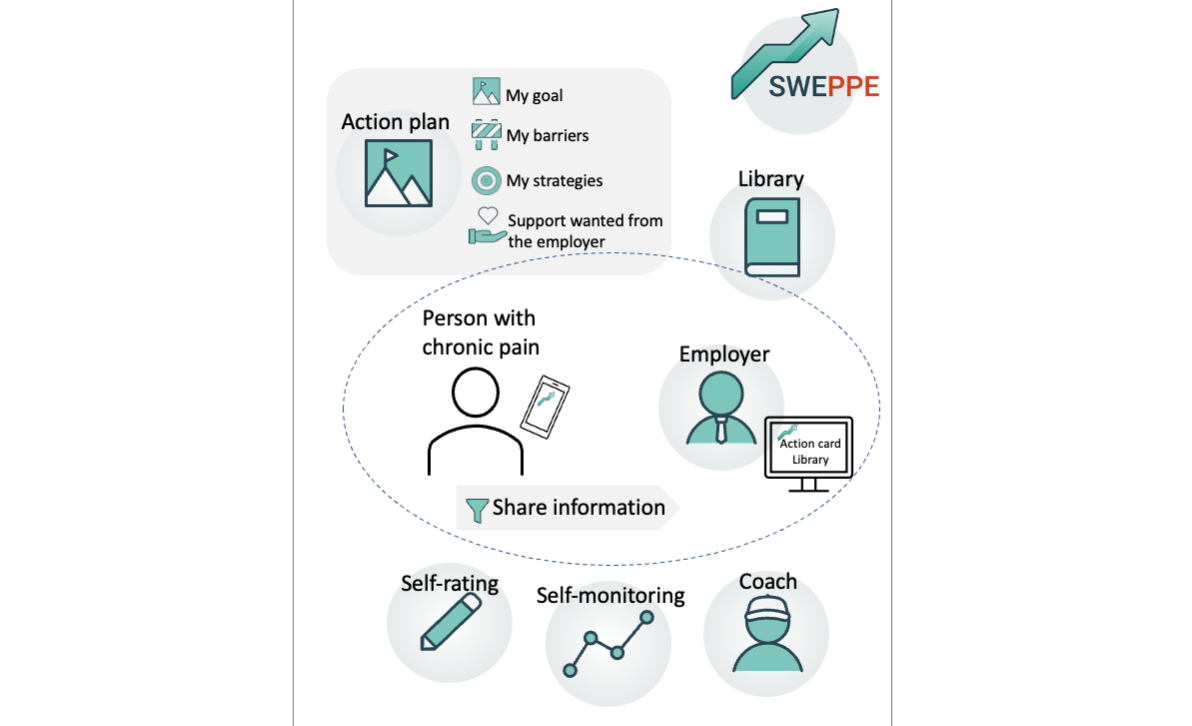
Overview of the functions in SWEPPE (sustainable worker, a digital support for persons with chronic pain and their employers).

The intervention consisted of the SWEPPE smartphone app. SWEPPE contains 6 modules: “the action plan,” “daily self-rating,” “self-monitoring,” “the coach,” “the library,” and “share information” (Figure 1).

Each module consists of different functions, and the user can choose to use one or several modules depending on the person’s needs. In the action plan, the person with chronic pain can set a work-related goal, identify barriers to RTW, develop strategies to handle the identified barriers, and support needed from the employer. Daily self-ratings of health or work aspects can be performed, and the user can choose which and how many aspects to rate. Self-monitoring consists of performing weekly evaluations of goal fulfillment, satisfaction, and work ability. Daily self-ratings and weekly evaluations are visualized using graphs. The library consists of informational texts and films about chronic pain and work. The coach function gives the person with chronic pain an opportunity to ask the coach a question and receive an answer in the app. The coach function is introduced through messages in the app. The coach is a group of occupational therapists with experience in clinical work and research on chronic pain. The module share information gives the person the possibility to invite and share information from the action plan with the employer and give the employer access to the library. The person decides who to invite and what to share on the app. If invited, the employer accesses SWEPPE via a web app and obtains access to the information the employee is willing to share [23]. The participants used SWEPPE for 3 months.

### Data Collection

Background data about the participants were collected using a questionnaire, which was filled out by 11 (69%) of the 16 participants. Data regarding how the 16 participants with chronic pain used SWEPPE were collected via the encrypted SWEPPE database safely stored at Linköping University and accessed only by the researchers and the technical staff. The data were collected by downloading the following information to an Excel (Microsoft Corporation) file:

- Action plan: weekly updates of the participants’ work-related goals
- Self-monitoring: the participants’ weekly evaluations of goal fulfillment, satisfaction, and work ability
- Self-rating: if daily registrations of at least one health aspect have or have not been performed, they are indicated by yes or no
- Self-rating: activity time distribution—daily registrations of time spent in different daily activities (paid work, household chores, activities with children, taking care of relatives or older adults, and participation in voluntary work)
- Coach function: if and when the participants have used the coach function during the intervention period
- Share information: if the participants have shared information with their employer, and if they did, what content they have shared from the action plan
- Support wanted from the employer: monthly updates of the type of supports the participants wanted from their employer

No data were collected about how the employers used the system.

### Data Analysis

Descriptive statistics were applied for the analysis of user data. The support the participants wanted from their employers was analyzed and grouped into categories based on similarities.

### Ethical Approval

This study was approved by the Swedish Ethical Review Board (Dnr 2020-01593).

## Results

### Use of the Modules in SWEPPE

The participants used the different modules in SWEPPE to a varied extent (Table 1).

**Table 1:** Overview of the use of each function on the group level (N=16).

Module and function		Total number of participants using each function, n (%)
**Action plan**		
	Setting a goal (yes)	11 (69)
	Identification of wanted employer support (number of registered employer supports)	15 (94)
**Self-monitoring**		
	Weekly evaluation of the goal and work ability (number of weekly evaluations)	11 (69)
**Daily self-rating**		
	Rating of at least 1 health or work aspect (number of registered days)	16 (100)
	Rating of activity time distribution (number of registered days)	14 (88)
**Coach**		
	Ask the coach a question (number of questions)	2 (13)
**Share information**		
	Share library and information from the action plan with employer (yes)	9 (56)

The 2 most frequently used functions were the daily self-rating of health or work aspects and the action plan. The coach function was the least used by the participants, with only 2 participants asking a question each to the coach.

The daily self-rating of at least 1 health aspect was used by all participants (N=16), with a median of 26 (IQR 8-87) daily registrations during the study period (Table 2).

**Table 2:** Frequencies of participants’ (N=16) use of the different modules in SWEPPE.

Participants’ sex	Action plan			Self-monitoring: weekly evaluation of the goal and work ability (number of weekly evaluations)^a^		Daily self-rating			Coach: ask the coach a question (number of questions)^b^
	Set a goal	Identification of wanted employer support (number of registered employer supports)^c^			Rating of at least 1 health or work aspect (number of registered days)^d^		Rating of activity time distribution (number of registered days)^e^		
Female	Yes	3	7		47		43	1	
Female	Yes	2	12		87		4	0	
Male	Yes	3	11		89		85	1	
Female	Yes	5	3		16		14	0	
Female	Yes	4	1		6		5	0	
Female	No	4	0		27		23	0	
Female	Yes	3	1		90		60	0	
Female	Yes	7	9		88		86	0	
Female	Yes	2	4		90		89	0	
Female	Yes	3	2		75		72	0	
Male	No	4	0		6		0	0	
Female	Yes	7	1		25		13	0	
Female	Yes	2	1		9		7	0	
Male	No	1	0		25		21	0	
Female	No	1	0		1		0	0	
Female	No	0	0		1		1	0	

^a^Median (IQR): 3 (1-8).

^b^Median (IQR): 1 (1-1).

^c^Median (IQR): 3 (2-4).

^d^Median (IQR): 26 (8-87).

^e^Median (IQR): 22 (9-69).

In the action plan, 15 (94%) of the 16 participants identified the support they wanted from their employers and 11 (69%) participants set a work-related goal. Of the 11 participants, 8 set the goal of working full-time and the remaining to work part-time. Self-monitoring was used by 11 participants who performed a median of 3 weekly evaluations of goal fulfillment, satisfaction with goal fulfillment, and rating of work ability during the 3-month study period (Table 2).

A total of 9 (N=16, 56%) participants shared information on SWEPPE with their employers, but for 3 (33%) of them, data were missing regarding what information was shared. The remaining 6 (67%) participants shared their goals, their strategies, and the support they wanted from the employer. In total, 5 (56%) participants also shared their barriers, and 4 (44%) participants also shared the graph showing the weekly evaluation of goal fulfillment, satisfaction, and work ability (Table 3).

**Table 3:** Summary of the type of information participants shared with their employers (n=9).

Participant number	Shared information (yes)	Type of information shared with the employer
1, 2, and 3	✓	Missing data regarding shared information
5, 8, 9, and 12	✓	“My goal”; “my barriers”; “my strategies”; “employer support”; and graph over the weekly evaluation of goal fulfillment, satisfaction, and work ability
6	✓	“My goal,” “my barriers,” “my strategies,” and “employer support”
7	✓	“My goal,” “my strategies,” and “employer support”

### Type of Support the Participants Want From Their Employers

A total of 15 (94%) of 16 participants identified the support they wanted from the employers on the SWEPPE app. The number of supports each participant identified ranged between 1 and 7, giving a total of 51 employer supports. These were sorted into 8 categories (Table 4).

**Table 4:** Overview of the support that employees wanted from the employers identified in SWEPPE by the participants (n=15)^a^.

Category	Values, n (%)	Type of support identified by the employees
Adapted work assignments	7 (47)	Not have to handle many things at the same time (then I might forget how I move, sit, or stand)
Adapted work assignments	1 (7)	Avoid walks demanding the load and transportation of objects
Adapted work assignments	1 (7)	Limit the amount of work in a very cold or hot environment or in an environment with a lot of noise or vibration
Work posture	4 (27)	To reduce or avoid challenging work postures (uncomfortable work postures or movements, sharply bent, stretched, or twisted, above shoulder height, or below knee height)
Work posture	1 (7)	Better work postures during shower and dressing
Work posture	1 (7)	To avoid or reduce uncomfortable hand grips, precision grips, repetitive flexion, or twisted movements in the arm or hand
Work posture	2 (13)	Opportunity to shift work posture regularly (stand, walk, or sit)
Work posture	1 (7)	To avoid repetitive movements
Breaks	8 (53)	Opportunity to take breaks and short rests
Work pace	5 (33)	To lower work pace and avoid stress
Work pace	2 (13)	Planning to reduce stress and create space for unexpected events
Work pace	1 (7)	To have more time for reading and working on assignments
Ergonomics	5 (33)	Access to an ergonomic workplace (chair, keyboard, mouse, or adjustable table)
Ergonomics	1 (7)	Continuous support regarding ergonomics
Workload	3 (20)	To reduce or avoid heavy lifts
Workload	1 (7)	Continued support with workload
Activity balance	1 (7)	Working at home once a week to reduce stimuli, preferably in the middle of the week, to restore energy
Activity balance	1 (7)	Schedule
Activity balance	1 (7)	Evenly distributed working h throughout the wk
Activity balance	1 (7)	More time for recovery
Knowledge and understanding from the employer	2 (13)	An employer who lets me do what I can and supports and pushes me
Knowledge and understanding from the employer	1 (7)	Understanding that some days my pain is so bad that I cannot go to work

^a^Data are reported as the number of participants reporting each type of support.

The 2 largest categories were support for adapting work assignments and work posture, which were identified by 9 (60%) of 15 participants. Not having to handle many things at the same time and helping to avoid uncomfortable work positions, hand grips, and repetitive movements were examples of support identified by several participants. The single most common type of wanted support identified by 8 (53%) participants was the opportunity to take breaks and short rest. The remaining categories covered help to adapt to work pace, have access to an ergonomic workplace, reduce workload, provide support related to activity balance, and increase knowledge and understanding from the employer of how pain affects their work ability.

## Discussion

### Principal Findings

This study describes the use of the digital support app SWEPPE by analyzing user data collected over 3 months from people with chronic pain who have participated in an IPRP. The results show that the participants used the different modules and functions in SWEPPE to a varying extent. The most frequently used function was the daily self-rating of health or work aspects, which all participants used to some extent. The part of the action plan most participants used was to identify the support they wanted from the employer, although many participants also set and evaluated a work-related goal. More than half of the participants shared information with their employers, and most of them shared their goals, their strategies, and the support they wanted from the employers. The participants identified a variety of support they needed from the employers, including adaptation of work assignments, work postures, breaks, work pace, ergonomics, workload, schedule, and increased understanding.

Self-monitoring of health aspects is a common way for people with chronic pain to manage their condition [27,28]. It is also a frequently used function in smartphone apps for people with persistent pain [29]. This is verified as daily self-rating on SWEPPE and was used by all participants. The self-rating and self-monitoring function on SWEPPE was also rated as one of the 3 most useful functions among participants in the development study of SWEPPE [23]. Studying user data provides a unique insight into the variation in how often self-rating was performed on SWEPPE, and not all participants performed registrations every day. The intention of the self-rating function on SWEPPE is to enable the user to identify connections between symptoms and behaviors to promote the development of positive habits. The reasons for how often the participants in the study chose to use the self-rating function on SWEPPE are not yet explored, but previous research has shown that the willingness to self-monitor among persons with chronic disease is not directly related to the perceived difficulties in daily activities. Rather, willingness to self-monitor health is related to the ability to control the condition and varies depending on the health condition to some extent [30]. O’Reilly et al [31] identified understanding the content, mastering technology, or a non–user-friendly design as barriers to using eHealth technology to control a health condition. As the self-monitoring of daily registrations on SWEPPE is a highly analytic task, it may not suit all people with chronic pain. However, all participants used this function to varied extents, which indicates an interest in following and monitoring their own health status.

The second most used function on SWEPPE was the identification of support the participants needed from their employers. The new findings in this study are the examples and the frequency of concrete needs and adaptations the participants required to reach their work-related goals. Support from the employer is a facilitator for successful RTW [9], and the categories in our study align with several types of support identified in the literature, such as changed or flexible working hours, extra time to complete tasks, or the possibility to change posture [2,4,9]. Making adaptations at the workplace can increase the employee’s margin of maneuver [32,33]; that is, the opportunity the worker must develop or influence altered ways of performing work tasks, considering the tools or methods that are provided at the workplace [33]. For people with chronic pain, it is vital to have options regarding how work assignments are performed, and the solutions must be tailored to the individual [8]. The support wanted from the employer identified on SWEPPE was shared by most participants who shared information with the employer. Interviews with some participants revealed that identifying employer support strengthened the participants requests for work adaptations [24]. This may also apply to those who did not share this information with their employers on SWEPPE. The examples of support wanted from the employers can be valuable for further examination regarding their usefulness for persons with chronic pain during RTW.

The function to set a work-related goal was used by most participants. In the literature, goal setting along with self-monitoring, is the most commonly used strategy for self-regulation and the promotion of health behavior change [34]. However, goal setting is uncommon on smartphone apps that target persistent pain [29,35]. Using goal setting can be one way to formulate what the person with chronic pain believes is achievable regarding work. However, change is not promoted only by setting a goal [36]; rather, commitment and work toward the goal are affected by several factors, such as motivation and self-efficacy [37]. Furthermore, Hennessy et al [34] concluded that using self-monitoring frequently can improve goal fulfillment. The participants using the function of setting a goal on SWEPPE also performed weekly evaluations of goal fulfillment and satisfaction with goal fulfillment, although the frequency of weekly evaluations varied among the participants. To what extent frequency of self-monitoring is related to fulfillment of work-related goals for persons with chronic pain using SWEPPE was not possible to analyze and needs to be studied in a larger sample. Furthermore, to achieve fulfillment of a work-related goal is not only dependent on the person with chronic pain and the ability to self-manage the condition. Rather, reaching the goal fulfillment of RTW is dependent on several factors, of which cooperation with the employer and other stakeholders is a fundamental part [9]. Thus, the intention of the function of setting and evaluating a goal and the option to share it with the employer on SWEPPE was to contribute to communication and interaction between the person with chronic pain and the employer. The decision to share information is made by the employees, and the reason for sharing data with the employer can depend on the relationship with the employer. The acceptability study revealed that individuals lacking a positive relationship with their employers were less likely to share information, whereas those who shared information found it easier to request and implement workplace adaptations [24]. The goals set on SWEPPE were shared with the employers by several participants, although not all shared their weekly evaluations. The sharing function is a unique feature on SWEPPE, and how this function can contribute to a successful RTW needs to be explored further. The labor market legislation in Sweden is robust and is designed to protect employees in general. However, the willingness to share information with an employer may vary in different contexts, depending on factors such as job security and an individual’s concerns about the potential consequences of reporting pain-related issues.

The aim of the coach function was to provide the user with someone to ask a question regarding self-management of chronic pain and their RTW process. However, the coach function was used by only 2 participants, asking a question each. However, the acceptability study of SWEPPE showed that those who did use it were satisfied with this function [24]. An explanation for the limited use of the coach could be the varying expectations of what a coach is. Therefore, this function needs further scrutiny. The varying frequency of how many of the different functions on SWEPPE were used reflects the flexibility SWEPPE is offering the user. Fernandes et al [38] showed that flexibility, allowing the individual to use a digital solution at their own pace, is a key enabler for engaging with the system. The need for the different functions in SWEPPE may vary over time depending on the individual’s situation, and the user can return to SWEPPE at a different time point to use some other functions. The participants used SWEPPE for 3 months, but living with chronic pain and creating a sustainable work situation is an ongoing process, and circumstances may change for the individual. Furthermore, the RTW or staying at work process involves not only the person with chronic pain but also stakeholders from different organizations [9]. Thus, the opportunity to use the different functions in SWEPPE can be one way of providing access to continuous support during the RTW process, which has been identified as an essential part of learning self-management of chronic pain [39]. However, it is necessary to continue studying the use of SWEPPE over a longer period to see if the use of different functions varies over time and how they contribute to supporting the individual.

The study findings can serve as valuable information for individuals with chronic pain, employers, and other stakeholders about how a digital support app can be used by the individual for support after IPRPs. These results underscore the significance of fostering collaboration between employees with chronic pain and employers while also promoting ongoing self-management of chronic pain. Indeed, the barriers raised by workers and the strategies proposed should be able to lead to improved measures to facilitate the RTW, can be transferred to similar situations in the future, and must be considered valuable knowledge for the employer and the labor market.

### Strengths, Limitations, and Future Directions

A strength of the study is the collection of user data from the SWEPPE database for analyzing the use of the different functions in SWEPPE. Reporting of patients’ adherence to eHealth–based self-management programs varies, and some studies have, for example, only reported the number of times the patient has logged in on a website or the number of sessions they have participated in [40]. Analysis of user data provides a more detailed picture of the clinical use of SWEPPE during the 3-month period. The limited sample size and the specific Swedish study context could impact the generalizability of the results, which therefore must be interpreted with care. Participant inclusion in the feasibility study may also be biased, as those who choose to participate may have a positive attitude regarding digital interventions. Furthermore, the small sample size does not allow for subgroup analyses, for example, based on sex or other participant characteristics. There was some missing data regarding shared information with the employer. Another limitation is the lack of user data on how the library was used or how the information shared by the participants was used by the employers. This type of information would increase our knowledge further regarding the use of SWEPPE and what interaction is enabled between the individual and the employer to create a sustainable work situation.

### Conclusions

In conclusion, the participants used multiple SWEPPE functions, such as daily self-registration, goal setting, self-monitoring, and employer support identification. This shows the flexible nature of SWEPPE, enabling individuals to select functions that align with their needs. Additional research is required to investigate the extended use of SWEPPE and how employers use shared employee information.

## Abbreviations

IPRP: interprofessional rehabilitation program
mHealth: mobile health
RTW: return-to-work
SWEPPE: sustainable worker, a digital support for persons with chronic pain and their employers
